# Design and Implementation of High-Availability Architecture for IoT-Cloud Services

**DOI:** 10.3390/s19153276

**Published:** 2019-07-25

**Authors:** Hyunsik Yang, Younghan Kim

**Affiliations:** School of Electronic Engineering, Soongsil University, Seoul 06978, Korea

**Keywords:** IoT service, high availability, IoT-cloud

## Abstract

For many vertical Internet of Things (IoT) applications, the high availability is very important. In traditional cloud systems, services are usually implemented with the same level of availability in which the fault detection and fault recovery mechanisms are not aware of service characteristics. In IoT-cloud, various services are provided with different service characteristics and availability requirements. Therefore, the existing cloud system is inefficient to optimize the availability method and resources to meet service requirements. To address this issue, this paper proposes a high availability architecture that is capable of dynamically optimizing the availability method based on service characteristics. The proposed architecture was verified through an implementation system based on OpenStack, and it was demonstrated that the system was able to achieve the target availability while optimizing resources, in contrast with existing architectures that use predefined availability methods.

## 1. Introduction

The Internet of Things (IoT) is promising for many new applications by enabling many objects around us to connect, communicate, and interact over the internet without human intervention. Huge data processing and data collection from sensors and other IoT devices are performed for those applications. Because IoT devices (sensors, actuators, etc.) are normally resource-constraint (i.e., storage and processing capabilities), sensor-cloud, or in other words, IoT-cloud [1,2,3,4,5] was proposed as a promising approach to address the limitations. IoT-cloud infrastructure constituting sensor networks and cloud is an expanded form of cloud computing for sensing services and IoT applications. In previous research [3,4,5], we proposed different models to integrate wireless sensor networks with the cloud for efficient IoT-cloud services. Such models consider the resource constraints of sensor devices [6,7,8] for sensor data collection as well as quality of service (QoS) for sensing services [3,4,5,9,10,11]. However, the previous works focus only on the communication between sensors and the IoT-cloud. In this paper, we expand our consideration into inside the IoT-cloud management architecture for high availability of IoT-cloud services.

For many vertical Internet of Things (IoT) applications (i.e., mission-critical IoT or industrial monitoring), the high availability is critical. In existing cloud systems, the system availability is usually improved by using various fault detection and recovery methods [12,13]. We observe that detailed fault detection and recovery operations depend on the requirements of the services provided by the cloud system, as well as available resources. However, how to implement an IoT-cloud system, which considers service characteristics and requirements to deploy corresponding appropriate fault detection and fault recovery schemes automatically, is a practical issue.

Fault detection can be divided into physical-, virtualized-, and application-level and may require some or all of them. The recovery method may also be different depending on the requirements of a service. The recovery methods of a cloud system can be classified into fault tolerance and redundancy methods [14,15]. The fault tolerance methods recover the faults by restarting the system or transferring it to another normal system. Redundancy methods, such as active–active/active–standby, improve a system by preparing a system that is the same as that for the provided service and using it as a replacement in cases of failure. Redundancy methods are faster than fault tolerance methods because they always prepare the same system as a backup or redundancy, but they have the disadvantage of consuming more resources.

In existing cloud systems for IoT, various studies on efficient service provision architecture and application placement methods have been conducted. However, existing availability methods are primarily designed for general cloud environments [9,10,11]. Alcaraz Calero et al. and Kim et al. [12,16] proposed a monitoring framework for physical and virtualization resources, ensuring availability for all services. Kourtis et al. [17] proposed an architecture that can detect faults in the cloud infrastructure and services.

In traditional cloud systems, services are usually implemented with the same level of availability. In such an existing cloud system, fault detection and fault recovery mechanisms are not aware of service characteristics. However, in the IoT-cloud, various services are provided with different service characteristics and availability requirements. A fault detection architecture that is dynamically based on various service characteristics has not been studied. For recovery, the backup system arrangement algorithm and the recovery architecture using the shared backup system are normally implemented. Jung et al. [15] proposed a method for improving service availability by using the active–standby method, whereas Zhou et al. [18] introduced an algorithm for effective placement of the backup system. However, a fault recovery architecture that is dynamically based on various service characteristics has not been implemented. Therefore, the existing cloud system is inefficient to optimize the availability method and resources to meet service requirements. To address that issue, this paper proposed a high availability architecture that is capable of dynamically optimizing the availability method based on service characteristics.

It should be noted that in an IoT-cloud environment where various services are provided, an architecture that is capable of dynamically optimizing the availability method based on service characteristics is required. An IoT-cloud environment that provides several types of services, such as V2V (vehicle-to-vehicle), Smart Grid, video surveillance, and health care should consider the environment and the characteristics of network services [1]. For example, a V2V service that transmits and receives data in real time may require a more precise fault detection method than other services. Such a service also requires the recovery time to be minimal. Therefore, to provide high availability for V2V services, multiple monitoring functions are required for the infrastructure and the applications. In such a case, a redundancy model that can reduce the recovery time may be more appropriate [19].

On the other hand, Botta et al. mentioned [1] Smart Grid services focus on data size and collection in a large-scale environment. In other words, the V2V service and the Smart Grid service have different characteristics. Each type of service may have a different traffic type, size, and data communication period. Therefore, the Smart Grid service may require a different fault detection or recovery method.

Furthermore, in environments where resources are limited, such as an edge cloud or a fog, an architecture that is capable of improving availability while optimizing resources by providing an appropriate failure detection and recovery function according to service characteristics is necessary.

In this study, an architecture that automatically configures fault detection and fault recovery methods according to the characteristics of IoT services in an IoT-cloud infrastructure is proposed. Based on the characteristics of an IoT service, the proposed architecture can provide the required availability in a resource-efficient manner by dynamically providing failure detection and a failover model through a template [19].

To verify the proposed architecture, it was implemented using OpenStack, and it was confirmed that it was able to provide the target availability. Furthermore, resources were optimized, as demonstrated by comparison with other architectures that use predefined fault detection and recovery functions.

The paper is organized as follows. In Section 2, related research on the features and availability of IoT-cloud is presented. In Section 3, the proposed architecture designed to dynamically detect recovery faults according to IoT services is described. Section 4 presents the implementation of the proposed architecture and experimental results. Finally, Section 5 concludes the paper.

## 2. Related Research

### 2.1. Characteristics of IoT-Cloud

IoT-cloud infrastructure [3,4,5,9,10,11,12] constituting sensor networks [6,7,8] and cloud is an expanded form of cloud computing for sensing services and IoT applications. Huge data processing and data collection from sensors and other IoT devices are performed for those applications. Because IoT devices (sensors, actuators, etc.) are normally resource-constraint (i.e., storage and processing capabilities), sensor-cloud, or in other words, IoT-cloud [20,21,22] was proposed as a promising approach to address the limitations. In our previous research [3,4,5], we proposed different models to integrate wireless sensor networks with the cloud for efficient IoT-cloud services. Such models consider the resource constraints of sensor devices [6,7,8] for sensor data collections as well as quality of services (QoS) for sensing services [3,4,5,9,10,11]. However, the previous research focuses on the communication between sensors and the IoT-cloud. In this paper, we expand our consideration into inside IoT-cloud management architectures for high availability of IoT-cloud services.

An IoT-cloud provides the management functions of IoT devices and applications for services. The IoT-cloud includes various services such as smart home, smart grid, and health care, and each application service has different requirements and environments depending on service characteristics. Moreover, it also has different requirements for availability depending on service characteristics [1].

In an IoT-cloud, certain availability mechanisms have been proposed, but they either provide mechanisms for specific types of data (such as collected data) or do not consider the characteristics of the cloud [23,24]. Recently, MEC (Mobile Edge Computing) architecture was introduced for IoT services. It provides IoT services at edge, so it improves performance, latency between IoT services and devices [20,21,22,25]. Compared to other types of clouds, an IoT-cloud should consider several environments owing to the variety of IoT devices (e.g., sensors or mobile devices), and each application should provide service for each IoT device.

Table 1 shows representative IoT services and their characteristics [1,26]. Service characteristics are classified according to the criteria defined below, and it can be confirmed that each service has different characteristics. The ‘o’ of an IoT service indicates that the IoT service has the corresponding property described in that column. Empty cells indicate that the IoT service does not have the property described in that column.

In the Table 1, latency means that this service should be ensured real-time data processing, and data processing means that this service requires high performance computing owing to high data throughput. Service continuity means that this service is stateful service or not (stateless), and data storage means that this service requires external data storage or not. Network size means the size of the network environment in which the service is provided and location refers to the cloud environment in which the service is located—in this paper, it is classified as core cloud and edge cloud. According to general characteristic of IoT service, we classified IoT service like Table 1 [1].

As the characteristics of an IoT service may vary, fault detection and fault recovery functions must be applied differently. For example, fault detection and recovery for services provided in an IoT-cloud environment may be adopted according to the service characteristics and the priorities of the targets to be monitored. One of the key features of smart metering services is to collect and visualize data received from various IoT devices [2]. Smart metering services normally do not require real-time response and processing; thus, they may allow more time than services requiring real-time response for detection and recovery. In addition, data processing and database failures are not as critical as application failures. Therefore, database monitoring is more important for availability, and fault recovery methods such as re-instantiation, respawn models may be more suitable than redundancy models. By contrast, a V2V service, which is used for communication between vehicles, requires real-time response and fast data processing. Therefore, such a service requires a redundancy recovery mechanism and multi-monitoring for fast detection and recovery.

In the case of a V2V application, a redundancy model is more suitable than other recovery mechanisms because it is faster. In addition, V2V applications are highly dependent on applications because they should quickly respond to and process data. Therefore, detection at the application level can facilitate expeditious fault detection [3].

A recovery mechanism can be adopted according to the cloud environment. For example, in the case of healthcare or CCTV services, which require real-time data processing, a redundancy model is suitable for fast recovery. However, in resource-constrained environments such as an edge cloud, resources may not be sufficient for configuring redundancy models. In this case, fault tolerance methods are not faster than a redundancy model, but the former offers better availability, and thus it should be preferred. Accordingly, the uniformized availability method may not be effective in an IoT-cloud providing various services. Therefore, a cloud architecture that can configure an optimized availability environment automatically according to various service characteristics is necessary.

### 2.2. Open Source Solutions for IoT-Cloud Availability

In Network Functions Virtualization (NFV) environments, ensuring availability is a challenging task. In the ETSI (European Telecommunications Standards Institute) standards, the Virtualized Infrastructure Manager (VIM) is responsible for controlling and managing the NFV Infrastructure (NFVI), e.g., computing, storage, and network resources, whereas the VNF manager (VNFM) is responsible for the lifecycle management of Virtual Network Function (VNF) instances. In this context, NFVI and VNFM should support a fault detection and recovery mechanism for life cycle management. Accordingly, in this study, an IoT-cloud availability architecture and procedure was designed on the NFV reference architecture. Thus, one of the representative cloud VIMs is OpenStack. OpenStack can control and manage resource pools. Several monitoring tools are proposed for fault detection, such as Ceilometer, Ngios, and Monasca [27,28]. These are also integrated with OpenStack to provide fault monitoring for the infrastructure. However, these solutions only consider errors caused by the NFVI and do not check the status of a VNF. Moreover, monitoring tools cannot support the recovery mechanism itself and should interwork with the cloud infrastructure to manage the status of the VNF or the NFVI. Furthermore, OpenStack has a Tacker project that develops the VNFM and provides a general VNFM function such as VNF CRUD (Create, Read, Update, Delete) [29]. Thereby, the basic IoT-cloud architecture can be implemented. However, a monitoring/recovery mechanism is required to detect failures. In an enterprise environment, several solutions are already used to check the status of the infrastructure. One of the more widely used monitoring tools is Zabbix [30]. Zabbix is an enterprise open source monitoring software for networks and applications that has been mainly used to manage faults in the physical server. Moreover, it is designed to monitor and track the status of various network services, servers, and other network hardware. It also provides a GUI so that users may easily check the status of the target on the website. However, its limitation is that it relies on an agent for obtaining monitoring data.

## 3. Proposed Architecture

In this section, the design for the proposed architecture is presented. An IoT-cloud service is composed of multi-layers, and it is necessary to configure the availability provisioning environment according to the service type. For that purpose, the proposed architecture was designed and implemented including the following elements. At first, the proposed architecture provided multilayer monitoring architecture for IoT-cloud and a common driver for extension of IoT-cloud monitoring function was also designed. In addition, the proposed architecture was implemented with OpenStack as a cloud infra and OpenStack Tacker as a management and orchestration (MANO).

**Figure 1:**
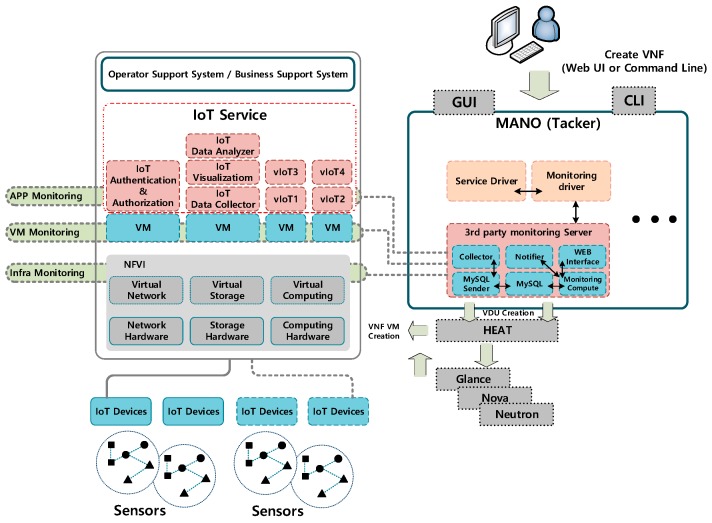
High-availability architecture for IoT-cloud.

As shown in Figure 1, the proposed architecture consists of two components. The left component is a cloud-based IoT service architecture. The right component is a management and monitoring architecture for IoT service and infrastructure. The former is the primary cloud environment that provides IoT services, which run as virtual network functions (VNFs), and has the role of receiving and processing data from IoT devices.

MANO (management and orchestration) is a top-level cloud management system that manages the entire infrastructure. In this study, an architecture providing availability in conjunction with MANO was developed. A monitoring driver interacting between monitoring tools and MANO was also designed. Furthermore, templates for automated monitoring and recovery were defined, and translators for availability templates were designed. Finally, a service driver supporting interworking with other monitoring tools was designed.

### 3.1. Architecture for IoT-Cloud Availability

**Figure 2:**
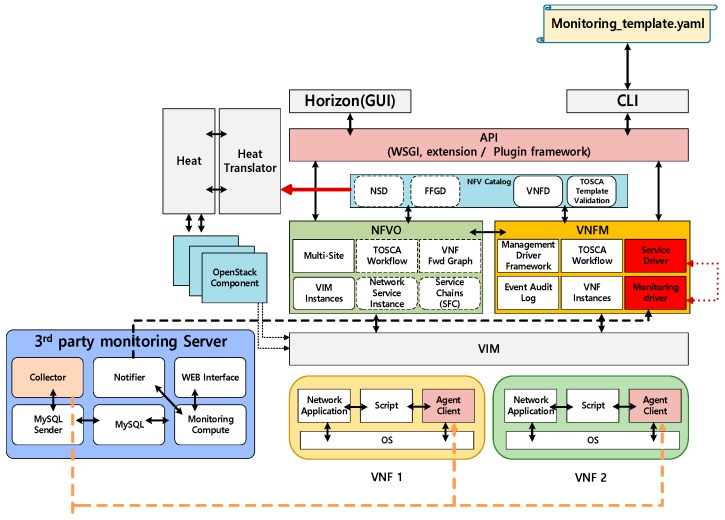
Design of components for IoT-cloud availability.

To provide availability for IoT-cloud services, an integrated architecture, which could interwork with other monitoring tools, was developed. The proposed architecture was designed using third-party monitoring tools and OpenStack Tacker (MANO). Figure 2 shows the availability architecture for IoT-cloud services.

In the proposed architecture, we designed the service driver, the monitoring driver, the monitoring server, and the translator which are placed in the controller as a software component. To communicate with each component, proposed component should be deployed Control Node. Monitoring server can be deployed on independent nodes, but communication with control nodes is essential. In this architecture, it is assumed that monitoring tools use agents to collect data from the target.

First, a monitoring driver that communicates with the monitoring server in MANO was designed. When an IoT service is created as a VNF, the driver sends VNF information to the monitoring server for the registration process. Another role of the monitoring driver is to handle the recovery action when an IoT service has an error.

Subsequently, a translator was designed to analyze the monitoring policy in the template. Its function is to translate the monitoring policy from the VNF descriptor (VNFD) and forward this information to the monitoring server. Tacker usually uses the VNFD, which includes VNF information, such as network configuration, images, size, or resources [31,32]; it also includes a monitoring policy. In the proposed system, the function of the translator was extended to recognize the monitoring function.

Finally, a service driver was designed. It calls a specific monitoring driver based on the VNFD. When the user requests fault management using specific monitoring, the service driver calls the specific monitoring function to check the status.

**Figure 3:**
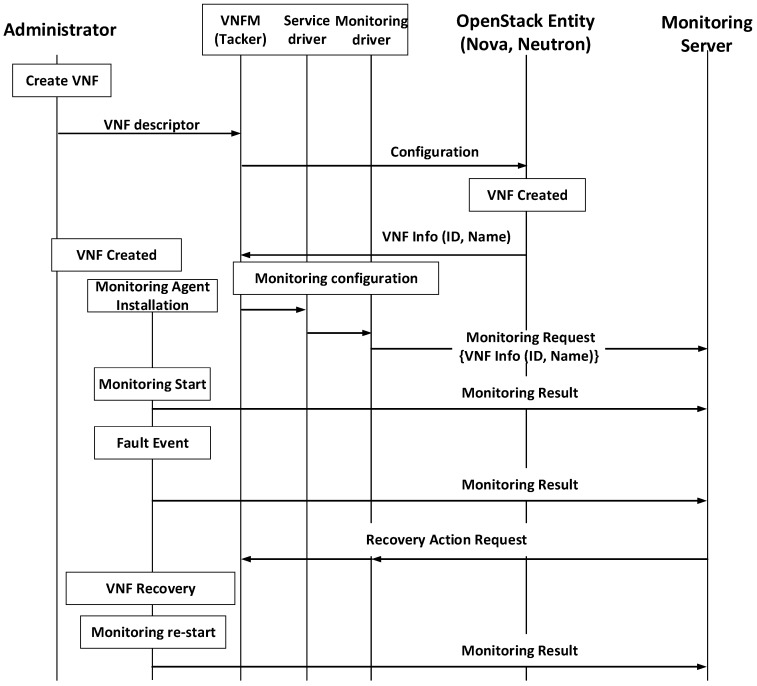
Procedure for service (Virtual Network Function (VNF)) fault management.

Figure 3 shows the procedures of the proposed architecture for IoT service availability. The administrator makes a request to the VNFM to create a VNF with a VNFD that includes the monitoring policy. When the VNFM receives data, it sends a request message to the OpenStack entity to create a VNF.

When the VNF creation is completed, the service driver reads the descriptor and finds the monitoring driver based on the information in the descriptor. Subsequently, the monitoring driver sends the monitoring request message with VNF information to the monitoring server (e.g., VNF id, monitoring policy).

In addition, the VNF begins to install the monitoring agent according to the script defined by the VNFD. This agent’s role is to collect the VNF information and forward it to the server. After installation, the agent periodically sends monitoring data to the server. When data meets the monitoring policy requirements, which are defined by the administrator, the monitoring server sends a recovery action request (e.g., rebuild, restart, or reconfigure) to the VNFM. For example, when an IoT service is launched in the cloud, the monitoring agent gathers monitoring data to check the status of the service (e.g., infrastructure or application status). When the monitoring server receives data from an IoT service VNF, and some resources are insufficient or the service fails, it requests a suitable recovery action to the VNFM based on the corresponding kind of failure.

### 3.2. Design of Monitoring Driver

**Figure 4:**
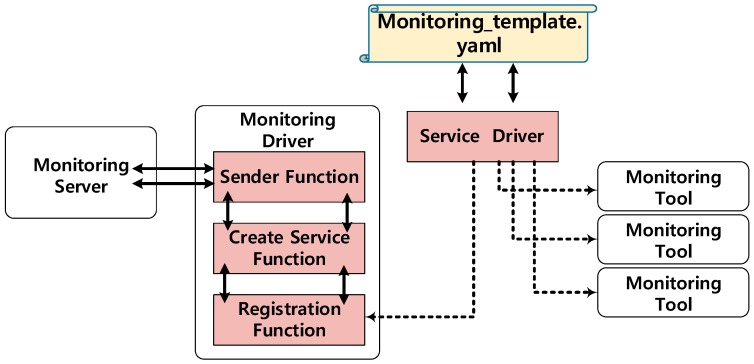
Components of monitoring driver.

Figure 4 shows the proposed design for the monitoring driver and service driver in MANO. The monitoring driver is composed of three small functions. The first function is the sender function, which adjusts the format when monitoring-related information is transferred to the monitoring server. All monitoring-related data is transmitted through the sender function. The create service function is the function which, after receiving the VNF information from MANO, extracts information related to the monitoring policy and forwards it to the sender function.

The registration function is used to register the specific monitoring driver as a monitoring function. This is a common function that communicates with the service driver. When the registration message comes from the service driver, this function registers the monitoring driver to the service driver. The service driver is a monitor call function that calls a specific monitoring driver based on the template. This function is designed so that other drivers can also be supported. Based on the monitoring parameter in the descriptor, the service driver calls a specific monitoring driver. For instance, if a user wants to use another monitoring driver, the service driver can register it to MANO as a monitoring driver.

### 3.3. Design Template for Availability

To provide the appropriate availability mechanism for IoT services, the monitoring and recovery functions should be provided automatically when a service is created. To support availability automatically, the fault detection and fault recovery policies should be included in the VNFD. In this respect, Tacker not only creates VNFs using TOSCA-based templates, but can also apply the availability policy to the VNFs. To enable this, the template was modified and the translator was extended. In the proposed architecture, a driver that can support other third-party monitoring tools was designed. Templates and translators for Zabbix were designed as a monitoring tool. The translator analyzes the content defined in the VNFD and converts it into a HOT template used by the cloud. To apply the monitoring policy defined in the proposed architecture, it is necessary to extend the translator.

**Figure 5:**
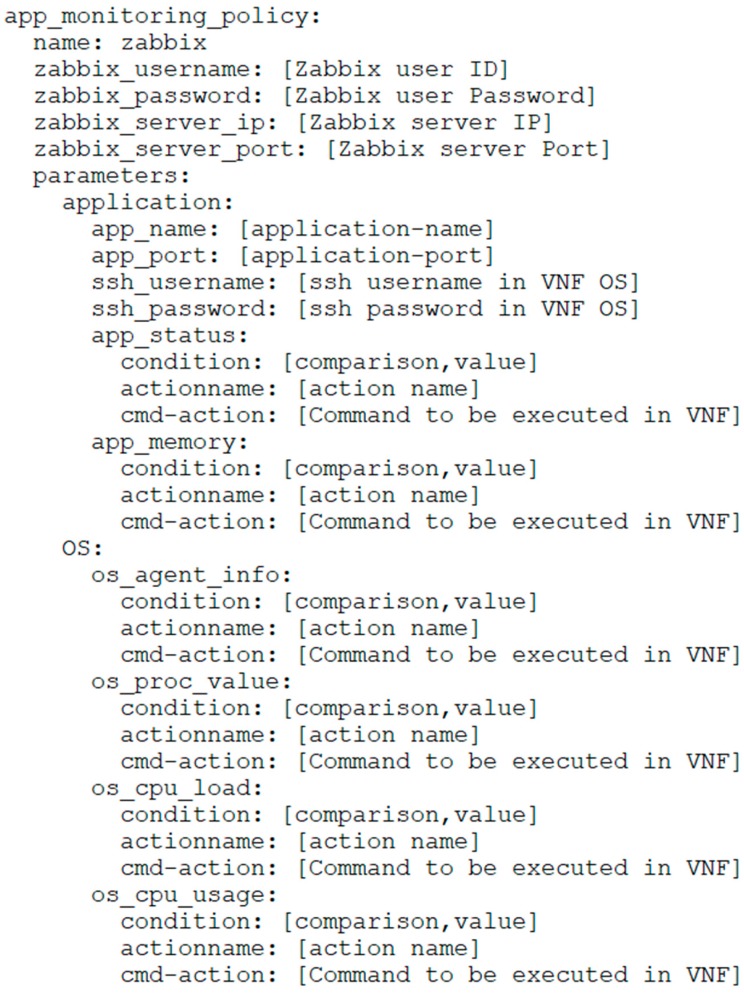
Virtual Network Function descriptor (VNFD) template for monitoring.

Figure 5 shows the VNFD for monitoring policy. The “app_monitoring_policy” part is composed of information about the target monitoring tool and account information for linking with the monitoring server, whence the monitoring driver requests registration from the monitoring server. The parameter part can be further divided into two parts: the application and the OS. The application part is used for defining a value for monitoring an IoT service, whereas the OS part is used for defining a value for monitoring infrastructure resources. The following procedures are added to provide availability:

The application part includes the monitoring policy for specific applications running on the VNF. It also includes account information of VNF connections and application information (e.g., port information) for checking the status. The “app-status” part includes information about the recovery policy for the VNF monitored, whereas “app_memory” includes a recovery policy according to memory usage, which is used by the application currently being monitored.

As shown in Figure 5, the OS part includes the monitoring policy for specific resources of a specific VNF or machine, such as CPU or memory. Specifically, “os_proc_value” indicates the number of process, “os_cpu_usage” indicates the CPU load, and “os_cpu_usage” indicates the I/O throughput.

The Zabbix monitoring driver uses an agent installed in the VNF. The agent collects data according to the monitoring policy and forwards it to the monitoring server. Currently, when a user uses Zabbix as a monitoring driver, the agent should be manually installed on each machine. However, as the number of monitoring nodes increases, installing agents on all nodes becomes difficult.

**Figure 6:**
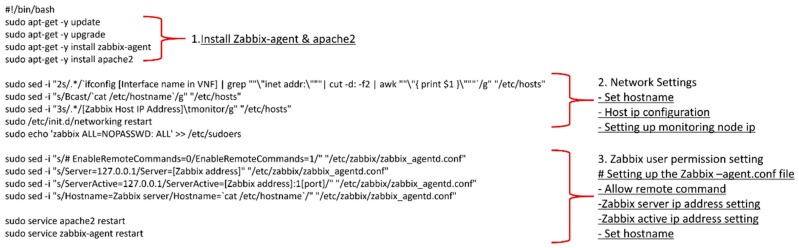
VNFD template for monitoring.

To resolve this, the agent installation was also defined in the VNFD. As shown in Figure 6, a user script was created in the user data part of the VNFD. Using the user data, VNF installs the agent, setups the network automatically for connection to the monitoring server, and thereafter the agent begins to collect the VNF data. Optionally, the user could also install the agent manually without the descriptor.

### 3.4. Discussion the Implementation Complexity

The new components proposed in this paper are a software component which installed into existing NFV architecture. They cannot be deployed to an independent physical node because that requires internal communication with the NFV management function (MANO) which is in the Control Node. In other words, any developer can install this architecture in their existing NFV Architecture and configure it. In addition, proposed architecture followed internal procedure of existing architecture, so the communication overhead and the control overhead of those components are as same as current existing architecture.

## 4. Implementation and Analysis

In this section, we introduce the implementation and verify the designed architecture. The proposed architecture is implemented using open source, and mathematical analysis and real performance verification are performed. We perform mathematical analysis with proposed architecture and verify each function such as the template operation, fault detection, and fault recovery function in the implementation environment.

### 4.1. Implementation

For evaluation, the proposed architecture was implemented in the environment described in Table 2. We used four servers that consisted of one controller and three computer nodes. Service drivers and monitoring drivers were deployed in the controller for interoperability with cloud management functions. In this paper, the monitoring server was placed in the controller but can be configured independently.

**Figure 7:**
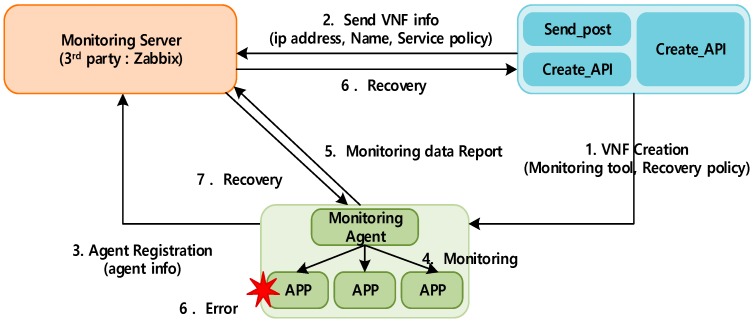
Procedure of service recovery for availability.

In this setting, a cloud environment and MANO functions were created. Figure 7 shows the scenario and the procedure to verify the proposed architecture.

The procedure for providing availability in this implementation is shown in Figure 7. A VNF was created using a Zabbix monitoring template, and an apache server was installed in the VNF. Subsequently, a simple IoT application was deployed on the VNF. The VNF creation, the monitoring driver registers the information of the created VNF with the monitoring server, and this is followed by agent installation, whereby the agent collects the data and forwards it to the monitoring server. In this scenario, the status of the IoT application running on the apache server is checked, and when there is an error, the agent notifies the monitoring server. After receiving notification from the agent, the monitoring server requests the VNFM to perform recovery of the VNF that has a fault. Note that in the proposed architecture, we designed the service driver, the monitoring driver, the monitoring server, and the translator as software components, which are placed in the controller node of the existing NFV architecture. Therefore, the architecture does not require any new physical node.

**Figure 8:**
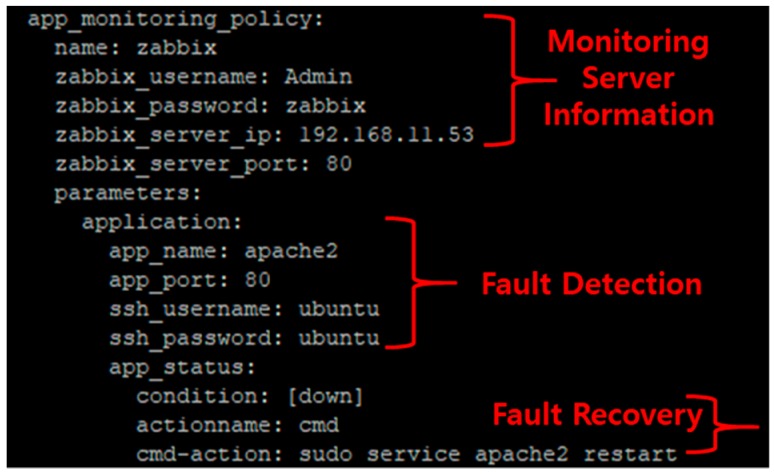
Example of VNF registration information.

Figure 8 shows an example of the fault detection and recovery method registered in the service according to the information defined in the template. Monitoring server information is registered according to the information defined in the template, which also includes a fault detection method and a fault recovery method; this information can be changed depending on the template.

**Figure 9:**
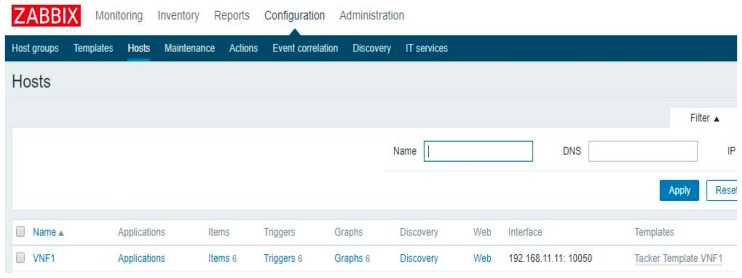
VNF registration on the monitoring server.

In Figure 9, the monitoring driver registers a VNF to the monitoring server. After creating the VNF, it runs the user data section defined in the VNFD. Upon installation, the agent collects the data and forwards it to the monitoring server. In this scenario, the status of the IoT application running on apache was checked.

**Figure 10:**
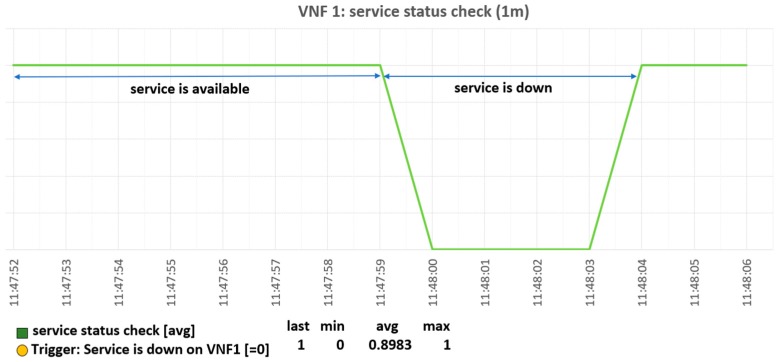
Monitoring result in the monitoring server.

Figure 10 shows a snapshot of the monitoring process for the IoT application. If the IoT application has an error, the agent notifies the monitoring server.

**Figure 11:**
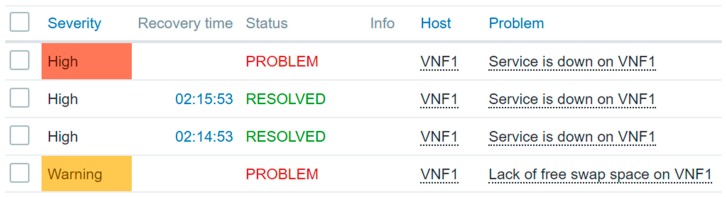
Monitoring result on the monitoring server.

When the monitoring server receives the notification from the agent (Figure 11) it requests the VNFM to perform recovery of the VNF that has a fault.

### 4.2. Resource Utilization Cost

This section presents a comparison of the proposed architecture with existing architectures. The total resource cost can be expressed as follows:

(1) $$C_{total}=C_{monitoring}+C_{recovery}$$

where $C_{monitoring}$ is the resource cost for monitoring and $C_{recovery}$ is the cost of recovery. $C_{total}$ is estimated as the sum of $C_{monitoring}$ and $C_{recovery}$. $C_{monitoring}$ is the total resource cost (CPU, RAM, and Network) required to monitor a single target and refers to single-server deployment [33,34]. It is derived as follows:

(2) $$C_{monitoring}=\frac{1}{1-u^{cpu}}\;\times \frac{1}{1-u^{net}}\;\times \frac{1}{1-u^{men}}$$

where $u^{cpu}$, $u^{net}$, and $u^{men}$ are the CPU, network, and memory utilization, respectively, of a VM on the same machine. $C_{recovery}$ represents the deployment cost of a VM that occurs depending on the recovery method and is given by [33,34].

(3) $$C_{recovery}\left(X\right)=e_{V\;}\overset{p}{\underset{i=1}{\sum }}\;\overset{m}{\underset{j=1}{\sum }}\left(X=\;\left(x_{ij}\right)\;,x_{ij}>0,\;i\;\in \;\left[1,\;p]\;and\;j\;\in \;[1,m\right]\right)$$

where $e_{V\;}$ is the fixed cost per deployed VM and $x_{ij}$ denotes the number of vCPUs assigned to VM i hosted on the physical host. For performance analysis, performance analysis environment was assumed that several different services ran on a single host, and 10% of the resources used by one service was allocated for monitoring. The assumption is based on the average percentage observed in our experiments. In addition, it was assumed that half of the VM required two monitoring methods and the remaining required one.

**Figure 12:**
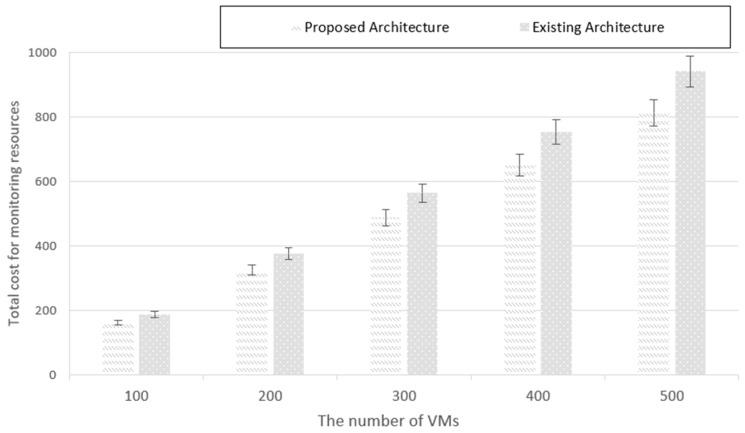
Comparison of monitoring resource cost.

Figure 12 shows that the monitoring resource cost of the proposed architecture is lower than that of the existing architecture. The gap increases proportionally to the number of VMs because the monitoring resource consumption increases. However, an overall the improvement is achieved up to 15%. In the existing architecture, two monitoring functions are allocated to all VMs regardless of the service, whereas in the proposed architecture, it is confirmed that the resource cost is reduced because a monitoring method specific to the characteristics of each service is provided.

**Figure 13:**
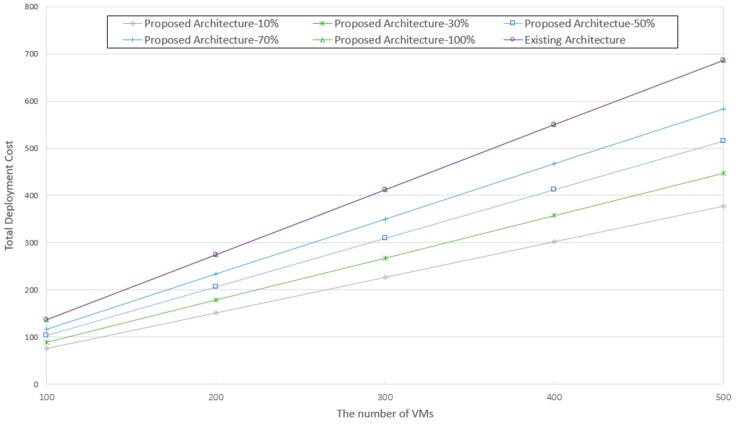
Comparison of recovery resource cost.

Subsequently, the resource cost for providing the recovery function was analyzed. Performance analysis was conducted considering typical environments where various recovery methods are necessary, such as an IoT-cloud. To investigate the change in cost owing to the environment, two recovery methods were used. Five cases were analyzed, where 10% (Case 1), 30% (Case 2), 50% (Case 3), 70% (Case 4), and 100% (Case 5) of the total services requested a redundancy method, and the remaining requested a respawning method. It was assumed that in all services, the same availability level was ensured regardless of the recovery method. As shown in Figure 13, it was confirmed that the proposed architecture reduced resource cost by approximately 2.5 times compared to the existing method. In the fifth case with 100%, the deployment cost is equal to that of the existing architecture. By providing the required recovery method for each service, the proposed architecture achieves better resource efficiency than the existing architecture. It was also observed that the proposed architecture was more cost-effective for more varied services compared to the existing method. The reason is that the proposed architecture can efficiently use resources and provide suitable availability methods for various service characteristics.

### 4.3. Performance Analysis

Herein, the performance of the monitoring and recovery functions in the proposed model are analyzed. To evaluate the performance of the proposed model, a VNF was created and the error detection time was checked in two cases: One is for detection of errors due to an increase in resource usage, and the other is for application errors. Furthermore, the number of applications was gradually increased to verify whether the proposed architecture is scalable. The basic testing scenario is as follows. We run simple IoT applications with VNFs. For monitoring, the system monitors infrastructure and application levels. For recovery actions, we perform application restarting and VNF respawning.

We conduct experiments with fifty IoT services used for testing, and the number of the applications for each IoT service was gradually increased from one to twelve. We used the application monitoring tool to detect application faults and ‘service restart’ as a recovery action

**Figure 14:**
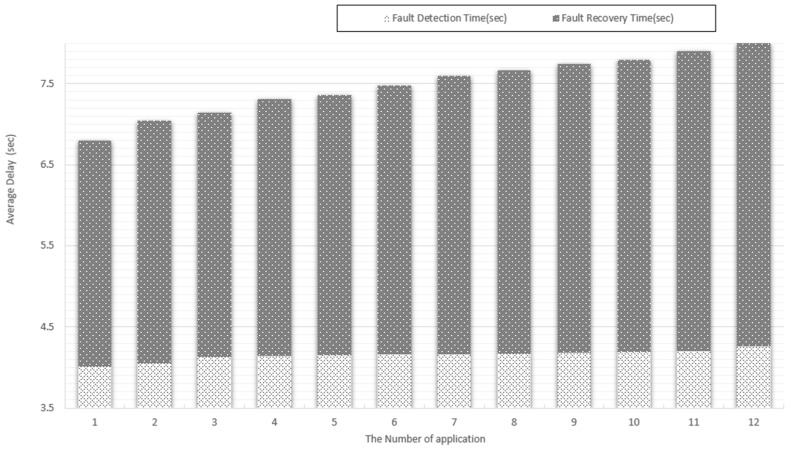
Multiple service fault monitoring performance.

Figure 14 shows the monitoring detection time and the recovery time according to the number of target applications (IoT services) in the configuration environment. The results show that increasing the number of applications does not impact the fault detection time significantly. The fault detection time difference between the case with one application and the case with seven applications is only 180 ms. The reason is that the proposed system monitors and detects faults quickly on the infrastructure and VNF level regardless of the number of applications installed in each VNF. However, the fault recovery time increases gradually proportional to the number of applications. The reason is that the higher the number of applications installed within a VNF the longer the time period is required to restart the VNF with all applications.

**Figure 15:**
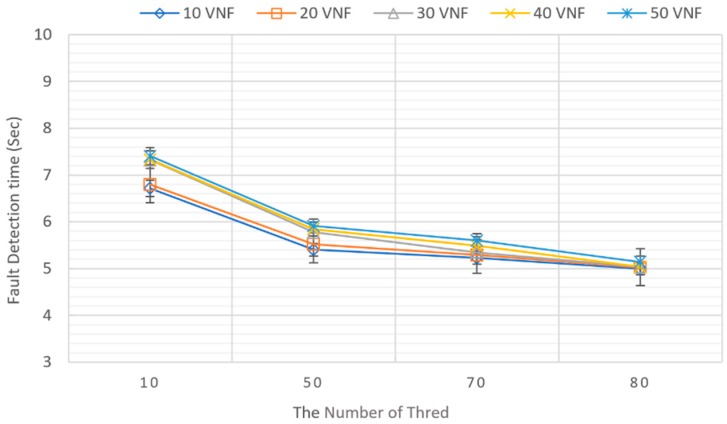
Resource fault monitoring performance.

Subsequently, experiments were conducted to check whether the monitoring server and the VNF manager performance are affected when the number of monitoring targets increases. The number of target IoT service nodes was gradually increased from one to 50, while the environment was maintained. In this experiment, CPU utilization was used as a resource parameter. In addition, the system was configured so that when the CPU usage rate for each IoT service node exceeds 70%, i.e., through increasing the number of threads, the system determines that a problem has occurred and should send a recovery action request to the VNFM according to the pre-recovery policy. Figure 15 shows the average detection time under various number of threads with different cases for the number of target IoT service nodes. The number of target nodes was increased from 10 to 50, and the number of threads was increased from 10 to 80 to vary the CPU load [35]. The experiment was run five times, and the average value was computed. The detection time of all cases is decreased gradually when the number of threads is increased. While the case with 10 VNFs witnesses the lowest fault detection time, the case with 50 VNFs shows the highest detection time due to the system workload. However, their detection time difference is small. The result indicates that the fault detection time does not increase significantly when we increase the number of VNFs. In our experiments, the results of all cases (10, 20, 30, 40, and 50 VNFs) are quite similar when the number of threads is 80 and over 80. We observed that with over 80 threads, the system processing is quick enough to process all cases similarly, so the detection time of all cases is similar.

**Figure 16:**
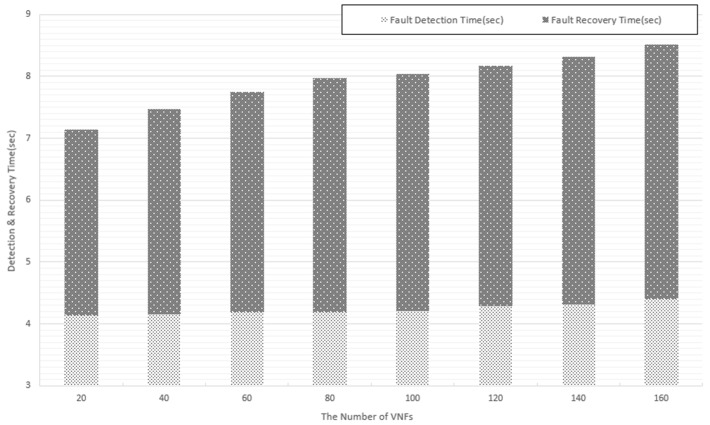
Application fault monitoring performance.

In the same environment using over 80 threads, we conduct experiments with application faults. We increase the number of VNFs from 20 to 160 to test the scalability of the system. Similar to Figure 15, the fault detection time in all cases in Figure 16 is quite similar. As explained in the previous experiment, when the system processing is quick enough, increasing the number of VNFs results in only a small increase in the fault detection time. In particular, when the number of VNFs is increased from 20 to 160, the fault detection time increases only approximately 400 ms. The result indicates that the system achieves a good scalability for fault detection. By comparing Figure 15 and Figure 16, we also find that the application fault can be detected faster, in comparison with the physical and virtualized level. The reason is that for the physical and virtualized level, our system has to monitor the resource usage until it reaches a certain level to conclude that a fault is detected. The fault recovery time increases proportionally when we increase the number of VNFs. The fault recovery action requires higher resource consumption and depends on the resource allocation to restart services, so the recovery time is impacted significantly by the number of VNFs sharing the same infrastructure.

In ETSI, availability is defined as mean time between failures (MTBF) and mean time to repair (MTTR) [14]. MTTR is the average length of the interval between repair points after a failure has occurred, and MTBF is the average length of working intervals without occurrence of any failures. Considering these definitions, availability can be expressed as follows:

(4) $$\mathrm{Availabilty}=\;\frac{MTBF}{MTBF+MTTR}$$

**Figure 17:**
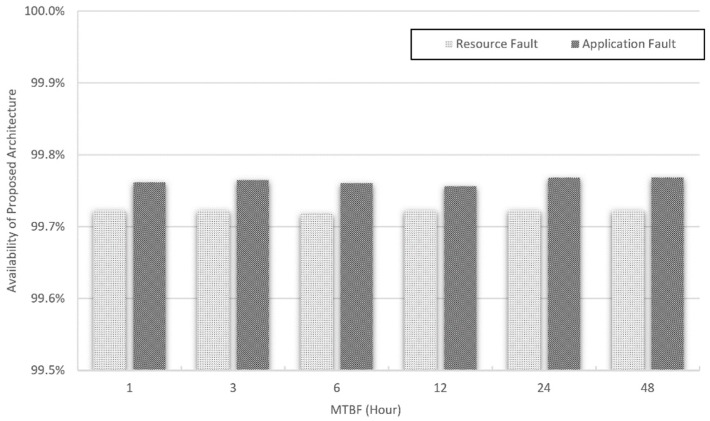
Availability of proposed architecture.

That is, if failure detection and recovery are executed quickly, the MTTR value is decreased, which can improve availability. Based on the availability expression, the proposed system was evaluated from 1 to 48 h. Errors were periodically generated every 30 min. Therefore, the value of the MTBF from 1 to 48 h and the experimentally obtained value of the MTTR were used. This experiment was conducted for resource and application monitoring. The results are shown in Figure 17.

The results demonstrate that the average availability in the proposed system is almost 99.80%. It was confirmed that the proposed system provides satisfactory availability in an NFV environment. It was also inferred that reducing the recovery and failure detection time affected the MTTR and MTBF.

## 5. Discussion

Through the analysis and experiments, results show the significant improvements of the proposed architecture compared to the existing architecture, in terms of the deployment cost and the monitoring resource cost, as well as the monitoring performance. The improvements are achieved because the proposed architecture is designed to automatically configure fault detection and fault recovery methods according to the characteristics of IoT services. In other words, depending on characteristics and requirements of IoT services once they are launched, the proposed architecture selects an appropriate model for fault detection and fault recovery, which not only satisfies the availability requirement, but also is efficient in term of cost. On the one hand, the existing architecture supports only predefined monitoring method and recovery method. It means that existing architecture provides the same methods for all kinds of services. This is inefficient as each service normally has a different requirement, as shown in Table 1. Through experiments, we also observed that the fault detection time is impacted significantly by the processing capability of the system but it only slightly depends on the number of applications. When the system processing is sufficient enough, the fault detection time of experiments is quite similar even when we increase the number of VNFs. On the other hand, the fault recovery time depends significantly on the number of applications and the number of VNFs.

In this paper, experiments are conducted with VM-based VNFs. For future research, we plan to extend the architecture to be implemented with the container environment. We are also interested in investigating the availability issue in a hybrid scenario where both VM-based VNFs and container-based VNFs are used. The reason is that VM-based VNFs and container-based VNFs have different characteristics. If a service chain is composed with both VM-based VNFs and container-based VNFs, the availability guarantee for the service chain is more complicated. This raises an interesting research question on how to optimally allocate resources and compose VNFs for a number of service chains that have different availability requirements.

## 6. Conclusions

A new architecture for providing availability for an IoT-cloud environment was proposed. The current architecture does not support appropriate fault detection and recovery. An architecture capable of automatically configuring the availability method according to service characteristics was designed and implemented. A template-based cloud architecture that can automatically configure fault detection and fault recovery methods subject to various service characteristics was proposed to ensure availability. Templates allow the application of the proposed method according to the characteristics of the service, and the feasibility of the method was demonstrated by comparison with the existing architecture. It was verified that the architecture provides optimal availability for IoT services. The analysis of the results demonstrated that the proposed system provides availability more dynamically and with higher efficiency compared to the existing architecture. Moreover, the proposed architecture was implemented, and its performance was verified. In future studies, we intend to research further on provisioning of availability in IoT-cloud continuously.

## Figures and Tables

**Table 1:** Internet of Things (IoT) service characteristics.

	Service Type	Latency		Data Processing		Service State		Data Storage		Network Size			Location	
Service Field		Critical	Non Critical	High	Low	Stateful	Stateless	Use	Non Use	Large	MID	Small	Edge	Core
Health care (heart beat)		O			O	O		O				O	O	
Smart home and smart metering			O		O		O		O			O	O	
Video surveillance		O		O			O	O		O			O	
Automotive and smart mobility		O		O		O			O		O		O	
Smart energy and smart grid			O		O		O		O		O			O
Smart logistics			O		O	O			O	O				O
Environmental monitoring		O	O		O	O		O		O			O	O

**Table 2:** Implementation specifications.

Entity	Condition	Version
Physical Server(4)	- Intel^®^ Xeon^®^ processor D-1520, Single-socket FCBGA 1667; 4-core, 8 threads, 45 W - RAM: 16 GB/Disk space: 256 GB - Controller Node (1)/Compute Node (3)	
Cloud OS	OpenStack stable	Queens
VNF Manager	OpenStack Tacker	Master

## References

[1] Botta A., de Donato W., Persico V., Pescapé A. (2016). Integration of Cloud computing and Internet of Things: A survey. Future Gener. Comput. Syst..

[2] Madria S., Kumar V., Dalvi R. (2014). Sensor cloud: A cloud of virtual sensors. IEEE Softw..

[3] Dinh N.T., Kim Y. (2018). An Efficient On-Demand Latency Guaranteed Interactive Model for Sensor-Cloud. IEEE Access.

[4] Dinh N.T., Kim Y. (2019). An Energy Efficient Integration Model for Sensor Cloud Systems. IEEE Access.

[5] Dinh N.T., Kim Y., Lee H. (2017). A Location-Based Interactive Model of Internet of Things and Cloud (IoT-Cloud) for Mobile Cloud Computing Applications. Sensors.

[6] Dinh T., Kim Y., Gu T., Vasilakos A.V. (2016). L-MAC: A Wake-up Time Self-Learning MAC Protocol for Wireless Sensor Networks.. Comput. Netw..

[7] Dinh N.T., Kim Y., Gu T., Vasilakos A.V. (2018). An Adaptive Low Power Listening Protocol for Wireless Sensor Networks in Noisy Environments. IEEE Syst. J..

[8] Dinh T., Gu T. A Novel Metric for Opportunistic Routing in Heterogenous Duty-Cycled Wireless Sensor Networks. Proceedings of the IEEE International Conference on Network Protocols.

[9] Mineraud J., Mazhelis O., Su X., Tarkoma S. (2016). A gap analysis of Internet-of-Things platforms. Comput. Commun..

[10] Vögler M., Schleicher J.M., Inzinger C., Dustdar S. (2018). Optimizing Elastic IoT Application Deployments. IEEE Trans. Serv. Comput..

[11] Xu Y., Helal A. (2016). Scalable Cloud–Sensor Architecture for the Internet of Things. IEEE Internet Things J..

[12] Alcaraz C.J.M., Aguado J.G. (2015). MonPaaS: An Adaptive Monitoring Platform as a Service for Cloud Computing Infrastructures and Services. IEEE Trans. Serv. Comput..

[13] Dinh N.T., Kim Y. (2019). An Efficient Reliability Guaranteed Deployment Scheme for Service Function Chains. IEEE Access.

[14] Jhawar R., Piuri V. (2013). Fault tolerance and resilience in Cloud computing environments. Computer and Information Security Handbook.

[15] Jung G., Rahimzadeh P., Liu Z., Ha S., Joshi K., Hiltunen M. Virtual Redundancy for Active-Standby Cloud Applications. Proceedings of the IEEE INFOCOM 2018—IEEE Conference on Computer Communications.

[16] Kim H., Yoon S., Jeon H., Lee W., Kang S. (2016). Service platform and monitoring architecture for network function virtualization (NFV). Clust. Comput..

[17] Kourtis M., Mcgrath M.J., Gardikis G., Xilouris G., Riccobene V., Rapadimitriou P., Trouva E., Liberati F., Trubian M., Batalle J. (2017). T-NOVA: An Open-Source MANO Stack for NFV Infrastructures. IEEE Trans. Netw. Serv. Manag..

[18] Zhou A., Wang S.G., Cheng B., Zheng Z.B., Yang F.C., Chang R.N., Lyu M.R., Buyya R. (2017). Cloud Service Reliability Enhancement via Virtual Machine Placement Optimization. IEEE Trans. Serv. Comput..

[19] Nabi M., Toeroe M., Khendek F. (2016). Availability in the cloud. State Art J. Netw. Comp. Appl..

[20] Li J., Jin J., Yuan D., Zhang H. (2018). Virtual Fog: A Virtualization Enabled Fog Computing Framework for Internet of Things. IEEE Internet Things J..

[21] Pan J., McElhannon J. (2018). Future Edge Cloud and Edge Computing for Internet of Things Applications. IEEE Internet Things J..

[22] Hesham E., Sharmi S., Mukesh P., Deepak P., Akshansh G., Manoranjan M., Chin-teng L. (2018). Edge of Things: The Big Picture on the Integration of Edge, IoT and the Cloud in a Distributed Computing Environment. IEEE Access.

[23] Zhu H., Huang C. Cost-Efficient VNF Placement Strategy for IoT Networks with Availability Assurance. Proceedings of the IEEE 86th Vehicular Technology Conference (VTC-Fall).

[24] Kumar A., Narendra N.C., Bellur U. Uploading and Replicating Internet of Things (IoT) Data on Distributed Cloud Storage. Proceedings of the IEEE 9th International Conference on Cloud Computing (CLOUD).

[25] Pavlos A., Eirini E., Papavassiliou S. Game-theoretic Learning-based QoS Satisfaction in Autonomous Mobile Edge Computing. Proceedings of the 2018 Global Information Infrastructure and Networking Symposium (GIIS).

[26] Gubbi J., Buyya R., Marusic S., Palaniswami M. (2013). Internet of Things (IoT): A vision, architectural elements, and future directions. Future Gener. Comput. Syst..

[27] Nagios. https://www.nagios.org/.

[28] Monasca. http://monasca.io/.

[29] Openstack Tacker. https://wiki.openstack.org/wiki/Tacker.

[30] Zabbix. https://www.zabbix.com/.

[31] VNF Descriptor. https://docs.openstack.org/tacker/latest/contributor/vnfd_template_description.html.

[32] Caroline Chappell (2015). Deploying Virtual Network Functions: The complementary Roles of TOSCA& NETCONF/YANG.

[33] Wood T., Shenoy P., Venkataramani A., Yousif M. Black-box and Gray-box Strategies for Virtual Machine Migration. Proceedings of the NSDI ’07: 4th USENIX Symposium on Networked Systems Design & Implementation.

[34] Yala L., Frangoudis P.A., Lucarelli G., Ksentini A. (2018). Cost and Availability Aware Resource Allocation and Virtual Function Placement for CDNaaS Provision. IEEE Trans. Netw. Serv. Manag..

[35] Stress-ng. http://kernel.ubuntu.com/~cking/stress-ng/.

